# Energy requirements for racing endurance sled dogs*

**DOI:** 10.1017/jns.2014.31

**Published:** 2014-09-30

**Authors:** John P. Loftus, Molly Yazwinski, Justin G. Milizio, Joseph J. Wakshlag

**Affiliations:** Department of Clinical Sciences, Veterinary Medical Center, College of Veterinary Medicine, Cornell University, Box 34, Ithaca, NY 14853, USA

**Keywords:** Canine nutrition, Athlete dogs, Energy requirements, DS, Dawson Yukon, ME, metabolisable energy, WH, Whitehorse Yukon

## Abstract

Endurance sled dogs have unique dietary energy requirements. At present, there is disparity in the literature regarding energy expenditure and thus energy requirements of these dogs. We sought to further elucidate energy requirements for endurance sled dogs under field conditions. Three sled dog teams completing the 2011 Yukon Quest volunteered to provide diet history. Nutritional content was evaluated and a mock meal was analysed for each team. Race data were obtained from www.yukonquest.com. Dogs were weighed at the start of the race in Whitehorse Yukon (WH), a mid-way checkpoint in Dawson Yukon (DS) and at the finish in Fairbanks Alaska. Data are average value per dog or per dog per d. Linear regression compared average weight loss to average kcal/dog consumed daily. Diets and feeding regimes were similar for all three teams. The average daily energy intake and nutrient content was similar for all diets. During leg one (WH to DS), team 1 gained weight overall, whereas the other two teams experienced weight loss. Linear regression revealed 37 638 kJ/dog/d (8995 kcal/dog/d) was required for weight maintenance. During leg two (DS to Fairbanks Alaska), average weights decreased for all three teams. The extrapolated kcal requirement was approximately 57 734 kJ/dog/d (13 799 kcal/dog/d). The carbohydrate contents of these diets also suggest that presumed fat intake for endurance sled dogs may be slightly less than previously thought. Finally, these data support the concept that dietary energy requirements vary substantially with additional variables such as load pulled, terrain and ambient temperature.

Endurance sled dogs have unique dietary requirements to maintain weight and provide energy for exercise^(^^1^^,^^2^^)^. It has long been recognised that their energy requirements are substantially higher during racing than when at rest by 2–5-fold. At present, there is disparity in the literature regarding energy expenditure and thus energy requirements of these dogs. Reported average daily energy expenditures using the doubly labelled water method range from approximately 16 700 kJ/dog/d (4000 kcal/dog) in one study^(^^3^^)^ to 47 100 (sd 5900) kJ (11 250 (sd 1409) kcal)/dog in another^(^^4^^)^. In the case of the former study, isotope-labelled water was administered subcutaneously, while it was given orally in the latter study. An important confounding factor using this method is the variation in naturally occurring ^2^H and ^18^O isotopes in water. In both studies, background samples were assessed and there were significant differences in isotope content of water available throughout the course of both races. Another factor that could account for differences between the studies is body weight, which was not reported in the second study. However, it is unlikely this would contribute a significant difference to energy requirements as most sled dogs are generally similar in size and body composition does not vary greatly in these athletes.

Substantial elevations in energy requirements for dogs engaged in endurance exercise are corroborated by other studies as well^(^^2^^)^. In the case of Inuit sled dogs undergoing training during the winter, energy requirements are as high as approximately 16 730 kJ/dog/d (12 000 kcals/dog/d)^(^^5^^)^. Other working dogs performing moderate or sprint activity require more modest increases in dietary energy. Kronfeld and colleagues suggested that open sprinting sled dogs required 16 736–20 920 kJ/dog/d (4000–5000 kcal/dog/d) through feed calculations during training and racing in similar conditions^(^^6^^)^. Hunting dogs in winter working for approximately 3 h a day require approximately 10 880 kJ/dog (2600 kcal/dog) daily during work^(^^7^^)^.

In addition to an increase in total energy requirements, the source of energy in the diet appears to be important in the sled dog. Dogs receiving greater than approximately 30–40 % of their metabolisable energy (ME) as carbohydrate can develop signs of ‘tying up’, coprophagy and hypoglycaemia during intense exercise that resolves when the proportion of ME from carbohydrate is reduced^(^^6^^)^. Thus, it has been recommended that high-performance sled dogs should receive a diet comprising 0–22 % of energy from carbohydrate^(^^4^^)^. Furthermore, diets high in fat and protein promote physiologic alterations that include higher serum concentrations of albumin, calcium, magnesium and NEFA in one study^(^^8^^)^ and cholesterol, glucose, lactic acid, NEFA and ketones in another^(^^9^^)^. In addition, muscle glycogen increases in sled dogs over time during endurance racing, suggesting a compensatory mechanism resulting in lower muscle glycogen depletion after an initial bout of activity^(^^10^^)^. Improvement in endurance performance related to a low-carbohydrate diet has also been reported in other breeds of dog such as Beagles^(^^11^^)^.

We sought to further elucidate energy requirements for endurance sled dogs under field conditions utilising diet history and diet analysis to assess average daily intake of protein, fat, carbohydrate and overall kilojoules per d for dogs taking into consideration changes in body weight during the race.

## Materials and methods

### Animals

All animal protocols were approved by both the Yukon Quest Board Ethical Committee and the IACUC of Cornell University. Four sled dog teams were initially enrolled in the study. One team did not complete the race. Three sled dog teams completing the 2011 Yukon Quest volunteered to provide diet history. Race data were obtained from www.yukonquest.com. Dogs were weighed at the start of the race in Whitehorse Yukon Territory, Canada (WH), a mid-way checkpoint in Dawson, Yukon Territory (DS), Canada and at the finish in Fairbanks, AK, USA. Dogs that did not complete the race were excluded from the study weights due to incomplete data.

### Diet analysis

Musher questionnaires were taken at the Dawson checkpoint and Fairbanks finish. The mushers were asked to give the total number of meat snacks fed per d, meals (amount of kibble and meat) fed and waterings (including the amount of meat used to ‘bait’ water which promotes consumption of water); as well as amount refused or discarded at each meal as an approximation. To ensure information was correct at the Dawson check point, the frequency and amount of commercial dog food used in meals and watering was assessed by weighing the kibble and meat in feed drop bags, and this was used to make an average meal. The samples of meat snacks were collected from the mushers and three of each type of meat snack (tripe, beef and chicken) were weighed on a gram scale and an average was obtained. This average was used to represent a ‘typical’ meat snack. Each type of meat snack was analysed (Dairy One) to assess crude protein, crude fat, crude fibre, sodium, potassium, magnesium, copper, iron, zinc and manganese. The nutrient information obtained from analysis was used to assess the ME from various substrates in the meat snacks. Data on ME, crude protein, crude fat, nitrogen-free extract and crude fibre of commercial dog foods used by the three mushers was acquired from the companies (Red Paw Power Edge 26K, Redpaw; Dr Tim's Dog Food, Momentum; Caribou Creek Dog Food, Caribou Creek Gold). The data from commercial dog food and meat snacks used were compiled utilising an excel spreadsheet into daily consumption of energy based on Atwater equations (16·7 kJ (4 kcal)/g of protein, 37·7 kJ (9 kcal)/g of fat and 16·7 kJ (4 kcal)/g of carbohydrate), total crude protein, total crude fat, total carbohydrate and crude fibre during the race^(^^12^^)^. Data were segregated for comparison into two halves of the race, first from WH to DS, then from DS to Fairbanks Alaska.

### Statistical analysis

Data are presented as the average value per dog per d unless indicated otherwise. Linear regression compared weight loss of each dog to average kcal per dog consumed daily. Regression data are presented as the best fit line ±95 % CI. Regression was considered significant with *P* ≤ 0·05. Data analysis was performed using Prism 6 software (GraphPad, Carlsbad, CA).

## Results

### Animal and race information

Each team comprised fourteen Alaskan husky crosses of similar size (mean starting weight ranging from 21·88 to 26·62 kg) at the start. All three teams that completed the race finished within 13 d. The average number of dogs per team from WH to DS was twelve for team 1, thirteen for team 2 and twelve for team 3. During the second half of the race the average number of dogs was ten for team 1, twelve for team 2 and eleven for team 3. The trail conditions were better in the first half of the race with only one area of mountainous terrain, more discernible and packed trails and ambient temperature ranged from −23 to −12°C. In comparison, trail conditions worsened in the second half of the race with two areas of mountainous terrain with higher elevations, less discernible trains (fresh blowing snow – poor footing) and lower temperatures (−40 to −18°C).

### Diets

Diets and feeding regimes were similar for all three teams. They consisted of commercial diets listed in the Materials and methods section and additional meat snacks provided as meals, in water during hydration and as raw meat snacks. Dogs on average for each team were fed three meals per d, provided two meat and kibble laden waterings, and between five and six meat snacks daily based on reported musher information. The average daily energy intake and nutrient content is depicted in Table 1 using Atwater equations based on calculation of crude protein, crude fat and carbohydrate fed from the meat and commercial dog foods fed. When assessing calculated meals from two of the three mushers to the analysed meals that were fed, there was approximately a 5 % variation in the fat and up to a 10 % variation in the protein *v.* calculated dietary composition, with per cent fat and protein being higher in analysed samples than in the calculated averages. This is presumably due to commercial reports from the dog food companies being data sheets from ‘typical’ analysis rather than actual analysis of these particular batches of food used. Based on calculations of manufacturer provided information and meat snack analyses, the total grams of protein in the daily diet was 532 g on average during the first half of the race and increased to 610 g in the second half. Average daily grams of fat and carbohydrate were 808 and 380, respectively, for the first half of the race. In the second half of the race, meals that were designed for twelve to fourteen dogs were fed to fewer dogs and the extreme conditions of the race led to more food being fed to each dog to help maintain body weight, thus the average grams of daily carbohydrate and fat increased to 934 and 455, respectively. More importantly, food refusals and discarded food was not reported by any mushers suggesting that all food was used on the trail and fed out during racing.

**Table 1.** Average daily energy consumption and nutritional intake per dog as per cent ME values based on Atwater values

	kJ	FAT (% ME)	PROTEIN (% ME)	CARB (% ME)	FIBER (g)
Whitehorse Yukon to Dawson					
Team 1	46 765	51	32	17	82
Team 2	30 589	47	38	15	51
Team 3	41 183	51	33	16	70
Average	39 510	50	34	16	68
Dawson to Fairbanks Alaska					
Team 1	54 840	51	32	17	99
Team 2	39 995	47	37	16	70
Team 3	43 857	51	33	16	76
Average	46 233	50	34	16	81

### Energy requirements

**Fig. 1.**
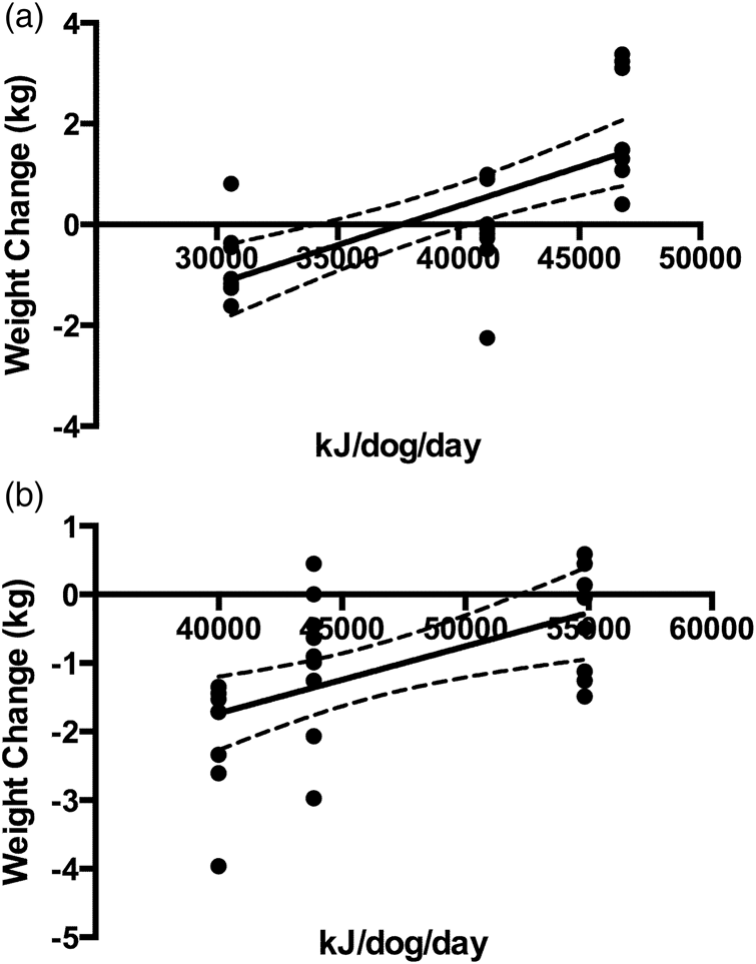
Energy requirements (kJ/d) for body weight maintenance. Changes in weight were compared with average team energy intake. (A) First half of the race, from Whitehorse to Dawson. For the first half of the race the average number of dogs included in the data set: team 1 *n* 12 dogs; team 2 *n* 13 dogs and team 3 *n* 12 dogs (*R*^2^ 0·5030; *P* < 0·0001). (B) Second half of the race, from Dawson to Fairbanks. For the second half of the race the average number of dogs included in the data set: team 1 *n* 11, team 2 *n* 12, team 3 *n* 10 (*R*^2^ 0·3030; *P* = 0·0036).

During leg one of the race, team 1 gained weight overall, whereas the other two teams experienced a mean loss in weight, even in the face of relatively favourable conditions. Linear regression revealed 37 638 kJ/dog (8995 kcal/dog) daily were required for weight maintenance, with a 95 % CI ranging from 34 112 to 40 484 kJ/dog/d (8153–9676 kcal/dog/d; *R*^2^ 0·5030; *P* < 0·0001; Fig. 1(A)). During the second half of the race, average weights decreased for all three teams. The extrapolated kcal requirement was 57 734 kJ/dog (13 799 kcal/dog) daily based on the linear regression analysis. The 95 % CI ranged from 52 206 to 71 049 kJ/dog (12 478–18 976 kcal/dog; *R*^2^ 0·3030; *P* = 0·0036; Fig. 1(B)) daily.

## Discussion and conclusion

Our data indicate that endurance sled dogs have an energy requirement similar to that described by Hinchcliff, where energy expenditure was 47 100 (sd 5900) kJ/dog/d (11 250 (sd 1409) kcal/dog/d)^(^^4^^)^. Interestingly, distance may only be one of the contributing factors in dictating daily energy expenditure and thus requirements for endurance sled dogs. When comparing the distances *v.* energy requirements/expenditures, the race in the present study was the longest at 1600 km (an average of 123–160 km/d for teams in the present study). In comparison, the race distances were 490 km (163 km/d) and 700 km (70 km/d) in the Hinchcliff and Decombaz studies, respectively^(^^3^^,^^4^^)^. Our data shows a substantial increase in energy required to maintain body weight during the second half of the race might be associated with terrain and ambient temperature, as the conditions during the race worsened at this time. Seeing as the load between the checkpoints and sled type do not change drastically while racing, a reduction in the average number of dogs per team during the second half of the race (i.e. fewer dogs pulling the load) provides an alternative or additional explanation for these results.

The present results must be interpreted cautiously since the present study relied strictly on musher interview/questionnaire regarding feeding during the race and extrapolated based on the average number of dogs fed per stage of the race. Although we asked mushers whether all meals were fed and the relative number of snacks at each stage of the race there may be reporting error. In the present study, ME was calculated from dietary analysis obtained from manufacturers, and chemical analysis of meat snacks fed using the Atwater equation^(^^1^^)^. In many instances, canine feed (processed diets) assessment is based on the modified Atwater equations, but due to the low-fibre contents of the proposed feeds and presumed high digestibility as well as the digestibility of raw meat we chose to use the Atwater equation, which provides a higher-energy designation to protein, fat and carbohydrates^(^^1^^)^. In addition, the information provided by the dog food companies are aggregate data of batches analysed providing crude protein and fat percentages that may be slightly lower and are likely the reason for the modestly higher protein and fat concentrations when compared with the calculated concentrations in the feed. In addition, we only measured the weight of the tripe, chicken and beef snacks used as an average of three weighed snacks from each meat type. This might not have been a true representation of the average meat-based snack used and the true average may have been higher, which may also be a reason for the slightly higher protein and fat concentrations in the analysed samples *v.* our calculations. Prior studies had used the doubly labelled water to measure energy expenditure based on the production of CO_2_ having incorporated the oxygen isotope administered as water, which is the gold standard^(^^3^^,^^4^^)^. However, results have been varied, which may suggest one or more confounding variables, including natural isotope abundance in drinking water, average distance travelled, etc. For instance, higher-energy expenditures corresponded to longer daily distance travelled when comparing the Decombaz (70 km/d) to the Hinchcliff study (163 km/d)^(^^3^^,^^4^^)^.

The energy from carbohydrate and fat of these diets may also suggest that fat intake for endurance sled dogs in the present study is lower than previously reported^(^^1^^,^^6^^)^. Previous data from Kronfeld suggest a diet with 66 % of energy from fat being superior to a diet with 45 % energy from fat^(^^6^^)^. The diets fed in the present study contained 47 % of energy from fat and 16 % of energy from carbohydrate, on average. This is certainly within the ideal range of 0–22 % energy from carbohydrate recommended, but is towards the higher end of this range^(^^1^^,^^6^^)^.

The disparity in protein content between calculated (6 and 10 % higher protein than calculated) and analysed nutritional profiles suggests a higher meat component to the diet given than what we calculated. Again, this may be from differences in the reported dog food quantities supplied to us from manufacturers. Furthermore, the average fresh meat addition might be slightly higher than what was reported to us. Given that fat and protein were slightly higher based on the analysis than what we calculated, then our carbohydrate content may in fact be slightly lower than reported here. Additionally, overall energy compared with that based on the Atwater equation could be different as well.

Overall, these results suggest that diet compositions among mushers are similar and provide appropriate macronutrient profiles for endurance work. The energy supplied in the first half of the race may in fact be adequate to maintain the endurance dog; however, the weight loss in the second half of the race suggests inadequate caloric intake. These finding may be important for the mushing dog community to understand feeding patterns in various field conditions allowing teams to fine tune-feeding practices to minimise weight loss while competing. Additionally, these results confirm the previous findings by Hinchcliff and colleagues suggesting that the energy requirements are likely in excess of 41 840 kJ (10 000 kcal)/d for endurance sled dogs in extreme racing conditions.
